# Comparison of outcomes in intrauterine insemination, *in vitro* fertilisation and intracytoplasmic sperm injection in men with and without varicocele

**DOI:** 10.7150/ijms.48005

**Published:** 2020-07-31

**Authors:** Xiao Li, Xiaoli Yang, Xianlong Wang, Li Wang, Jiaolong Liu, Feifei Cai, Yaqing Wang, Shaoming Lu

**Affiliations:** 1 Center for Reproductive Medicine, Shandong Provincial Hospital Affiliated to Shandong University, National Research Center for Assisted Reproductive Technology and Reproductive Genetics; The Key Laboratory for Reproductive Endocrinology of Ministry of Education, Jinan, Shandong 250021, P.R. China.; 2 The Medical Scientific Research Center of Guangxi Medical University, Nanning, 530000, P.R. China.

**Keywords:** varicocele, IUI, IVF, ICSI

## Abstract

**Objectives:** To investigate the differences in clinical pregnancy, miscarriage, and live birth rates when male partners were diagnosed with a varicocele and to compare these outcomes to those without and study the outcomes based on the grade of varicocele.

**Methods:** The retrospective study was based on a cohort of consecutive infertile couples undergoing assisted reproductive technology (ART) at the Reproductive Center of Shandong Provincial Hospital affiliated to the Shandong University during the period between January 2017 and December 2018. A total of 4203 couples comprised of men with and without varicocele undergoing the first ART cycle (1501 intrauterine inseminations (IUI), 1623 *in vitro* fertilisations (IVF) and 1079 intracytoplasmic sperm injections (ICSI)) were included. Semen parameters and ART outcomes were determined.

**Results:** ICSI (26.5%) originated from men with a significant lower level in sperm concentration and motility but with a strict normal morphology had a higher prevalence of varicocele than men undergoing IUI (20.7%) and IVF (18.1%). In IUI, the odds ratios (ORs) for pregnancy and live birth were significantly lower for couples in men diagnosed with grades 1 or 2 varicocele as compared to those for men with grade 3 varicocele. In IVF, ORs for live birth where men were diagnosed with grades 1 or 2 varicocele were also lower than those for men with grade 3,whereas a higher miscarriage rate was found when men had grades 1 or 2 varicocele than when men had grade 3. However, for ICSI, no significant outcomes were found in grades 1, 2 or 3 varicocele versus the no varicocele group.

**Conclusions:** The increasing grade of varicocele was negatively associated with sperm parameters and can alter the outcome of further IUI/IVF.

## Introduction

Varicoceles are defined as dilated, tortuous spermatic veins in the pampiniform plexus of the scrotal veins and are associated with male infertility. Varicoceles are observed commonly among infertile men and have been associated with abnormalities in semen analyses. They are found in approximately 10-20% to 30-40% of the general and infertile population, respectively, and have long been recognized as a common cause of infertility 1, 2, 3.

Varicoceles can result in an overall impairment of sperm production characterised by abnormal semen quality and a reduced fertilising capacity of the male gamete 4,5. They are associated with testicular volume loss and endocrine abnormalities and can also increase the intratesticular pressure, leading to an attenuation in blood flow and resultant hypoxia and an increased testicular temperature 6,7. In addition, toxic metabolites originating from the adrenal glands can reflux and damage the sperm DNA, causing abnormalities in the hormone profile 8.

In men with persistent varicocele-associated infertility, assisted reproductive technologies (ARTs), such as intrauterine insemination (IUI), *in vitro* fertilisation (IVF), and intracytoplasmic sperm injection (ICSI), can either serve as an alternative to surgery or as an adjuvant therapy to achieve pregnancy 9, 10. However, despite the increasing use of ART for male infertility, few studies have addressed the specific usefulness of assisted reproductive techniques in men with varicocele.

Given the unusual sperm abnormalities and the potential adverse effect of ART in men with varicocele, it is of interest to determine if ART outcomes using these sperms differ from those with no varicocele. The chief goal of the present study was to look at the fertilisation, pregnancy, and miscarriage rates when using sperm from men with and without varicocele. The groups were stratified according to the grade of varicocele to correlate outcomes with the severity of the disease.

## Materials and Methods

### Study population

This retrospective study was based on a cohort of consecutive infertile couples undergoing ART at the Reproductive Center of Shandong Provincial Hospital affiliated to Shandong University during the period from January 2017 to December 2018. A total of 4203 couples undergoing the first ART cycle (1501 IUI, 1623 IVF, and 1079 ICSI) were included. The study was approved by the institutional research ethics board. All patients were counselled and signed a consent form approved by the local ethics committee. The criteria for evaluation of infertility included a period of minimum one year of unprotected intercourse. The inclusion criteria for the female partner were (i) age <40 years; (ii) body mass index (BMI) <30 kg/m^2^ and (iii) baseline follicle-stimulating hormone (b-FSH) <12 IU/l.

The presence of varicocele was evaluated by inspection and palpation by trained physicians according to a standardised protocol for physical examination. Varicocele was graded with the men in a standing position (Dubin grading system) as grade 1 (only palpable during the Valsalva procedure), grade 2 (palpable in the resting state), and grade 3 (plainly visible) 11. Semen samples were collected by masturbation and measured according to the WHO guidelines (World Health Organization, 2010).

Regarding demographic data including male/female age, female b-FSH, sperm parameters, number of eggs retrieved, number of good embryos, fertilisation rate, good embryos rates, pregnancy rates and live birth rates. The choice of fertilisation method was based on the infertility diagnosis. While couples diagnosed with unexplained infertility were referred to IUI, the IVF group mainly consisted of couples with female infertility. The criteria for performing ICSI was a total sperm count of <500 000 after gradient centrifugation.

### ART procedures

In patients undergoing IUI, all hormone stimulation and insemination procedures were performed as previously described 12. In patients undergoing IVF/ICSI, hormonal treatment, oocyte retrieval, gamete handling and culture, and embryo transfer were performed as previously described 12.

Clinical pregnancy was defined by the presence of a gestational sac with heartbeat observed by ultrasound scanning at 5-7 weeks after embryo transfer through ART. Live birth was defined as delivery of any viable new-born at 28 weeks or more gestation after ART. Miscarriage was defined as any spontaneous interruption of clinical pregnancy. Ectopic pregnancy was defined as a pregnancy that developed outside the uterine cavity. Biochemical pregnancies were not included in the analysis.

### Statistical analysis

The subjects were divided into no varicocele and grades 1-3 varicocele according to the presence and grades of their varicocele. Men with bilateral varicocele were graded according to their highest grade of varicocele.

SPSS +16.0 software (SPSS, Chicago, IL) was used for statistical analyses. Continuous variables were presented as mean and standard deviation and categorical variables were presented as frequencies and percentages. Bivariate analyses were run to test between-group differences (with and without varicocele) using two-sided t-tests for continuous variables and chi-square or Fisher's exact tests for categorical variables.

For each of the three treatments (IUI, IVF, and ICSI), odds ratios (ORs) with 95% confidence intervals (CIs) for main ART outcome variables were estimated for high grade 3 compared to those without a varicocele using the binary logistic regression adjusted for confounders. The rationale for this was based on previous studies in which men with grade 3 varicocele seemed to have a lower live birth rate than those without varicocele in IUI and IVF cycles. This was also done for grades 1 and 2 in IUI, IVF, and ICSI cycles. Furthermore, couples treated with ICSI were compared to those treated with IVF with respect to main ART outcome variables within the grade 3 group. Male age, male BMI, female age, female b-FSH, sperm concentration, sperm motility, sperm normal morphology, fertilisation rate, good embryos rate, and embryos transferred were considered as potential confounders. All tests were two-tailed and the level of statistical significance was set at 0.05.

## Results

### Varicocele data

In the total 4203 cycles included in the study (IUI: 1501, IVF: 1623, and ICSI: 1079), 891 males (21.2%) with varicocele participated in ART, including 311 (20.7%), 294 (18.1%), and 286 (26.5%) males participating in IUI, IVF and ICSI, respectively. Although no significant difference in the prevalence of varicocele and sperm parameters was observed in men participating in IUI and IVF treatments (*p*>0.05), men participating in ICSI with a significant lower sperm concentration, motility, and strict normal morphology had a higher prevalence of varicocele than men participating in IUI and IVF (*p*<0.01).

Out of 891 males with varicocele, 788 men (88.4%) had unilateral left-sided varicocele, and 103 men (11.6%) had bilateral varicocele. According to the varicocele grade, 478 (53.6%), 231 (25.9%), and 182 men (20.4%) had grades 1, 2 and 3 varicocele, respectively. The results are shown in **Table 1.**

### IUI

Sperm concentration and strict normal morphology were lower in the grade 3 (49.39±15.83 million/mL and 2.31±0.78%) than in the no varicocele group (74.94±38.49 million/ml and 3.11±1.61%), corresponding to a 34% relative reduction. These parameters corrected negatively with the presence of grade 3 varicocele (OR: 0.988; 95%CI: 0.980-0.996). Sperm motility was lower in the grade 3 than in the no varicocele group but not significantly (**Table 2**).

**Figure 1:**
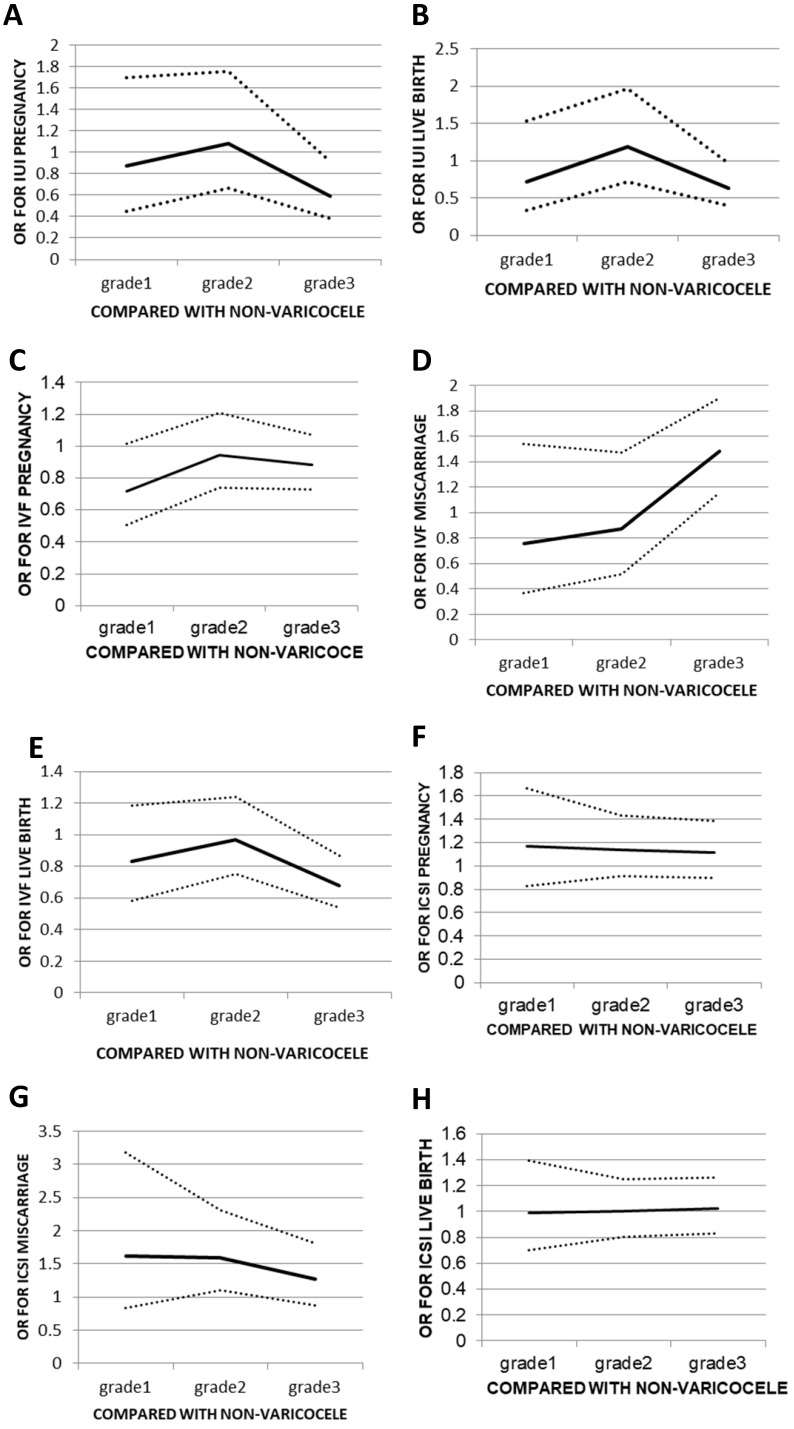
** Odds ratios (ORs) for different outcomes of assisted reproduction treatment in relation to non-varicocele group.** (**A**) pregnancy following IUI; (**B**) live birth following IUI; (**C**) pregnancy following IVF; (**D**)miscarriage following IVF; (**E**) live birth following IVF; (**F**) pregnancy following ICSI; (**G**) miscarriage following ICSI; (**H**) live birth following ICSI. Data are OR (±95% CI).

When comparing grades 1, 2, or 3 to the no varicocele group, there was no significant change in OR for grades <3. The OR was 0.871 (95% CI: 0.448-1.695) for grade 1, 1.083 (95% CI: 0.669-1.752) for grade 2, and 0.593 (95% CI: 0.383-0.918) for grade 3. The same trend was seen for the live birth rates from 0.718 (95% CI: 0.335-1.538) for grade 1 and 1.192 (95% CI: 0.721-1.971) for grade 2 down to 0.628 (95% CI: 0.399-0.969) for grade 3 (**Figure 1A, B**). Lower pregnancy and live birth rates were seen when male partners had a grade 3 varicocele compared to those seen when male partners did not have varicocele (4.6% vs. 14.4%, p<0.05 and 4.6% vs. 12.4%, p<0.05, respectively). Outcomes such as miscarriages and ectopic rates in IUI did not have a sufficient number of events to allow for a statistical analysis to be performed (**Table 3**).

### IVF/ICSI

In IVF, although sperm parameters were lower in the grade 3 group than in the no varicocele group, no statistically significant differences were seen between grades 1, 2, or 3 groups versus the no varicocele group with respect to sperm concentration, motility, and strict normal morphology. There were also no statistically significant differences between the grade 3 and no varicocele groups in terms of the fertilisation rate or the good embryo rate in IVF (**Table 4**). However, a significantly higher chance of obtaining a miscarriage and a lower live birth rate was seen in the grade 3 varicocele group compared with those in the no varicocele group. Women who had a live birth tended to be younger (30.68±3.89 years) than women who did not conceive (31.63±4.20 years, *p*<0.05) (**Table 3**). Compared to the no varicocele group, no significant change in miscarriages rates was found in IVF when the grade of varicocele was below 3, but the OR increased from 0.758 in grade 1 to 0.871 in grade 2 and 1.479 in grade 3, corresponding to a 41.1-48.7% increase. A similar pattern was seen for the live birth rates in IVF, with ORs of 0.833 (95% CI: 0.585-1.186), 0.967 (95% CI: 0.753-1.242) and 0.681 (95% CI: 0.537-0.865) for grades 1, 2, and 3, respectively, corresponding to a 22.3-42.0% decrease (**Figure 1C, D**).

In ICSI, no statistically significant differences were seen in fertilisation or good embryo rates in grades 1, 2 or 3 versus the no varicocele group; the same was true for pregnancy, miscarriage, and live birth rates (**Tables 3 and 5**). When comparing grades 1, 2, or 3 in relation to the no varicocele group, there were no significant changes in OR for pregnancy or live birth rates in ICSI. The ORs of pregnancy and live birth rate for grades 1, 2 and 3 were 1.173 (95% CI: 0.826-1.666), 1.142 (95% CI: 0.0.910-1.432), and 1.115 (95% CI: 0.897-0.385) and 0.990 (95% CI: 0.701-1.396), 1.001 (95% CI: 0.803-1.248), and 1.025 (95% CI: 0.631-1.265), respectively (**Figure 1E, F**).

## Discussion

In this largest ever-reported study of 4203 infertile couples, the presence of varicocele was associated with poorer semen quality in IUI/IVF. In accordance, the concentration of the spermatogenesis-related ICSI treatment was also associated with a higher presence of a varicocele than in IUI or IVF. Lower pregnancy and live birth rates were observed when male partners had a grade 3 varicocele when compared to the no varicocele group in IUI. They also had a higher miscarriage and lower live birth rates in comparison to the no varicocele group in IVF. These results indicated that poorer IUI or IVF outcomes were associated with varicocele.

Varicocele has been associated with impaired spermatogenesis, even if the mechanisms behind this impairment are still not fully clear. In 1992, a large multicentre study reported the semen parameters of over 7000 patients, of whom 1253 had varicocele. This condition was found in 25% of patients with impaired semen parameters and 12% of normozoospermic patients 13. Moreover, some authors have suggested a dose-dependent effect of varicocele on male reproductive potential, asserting that subjects with a high-grade or bilateral varicocele would present progressively worse semen parameters. Vivas-Acevedo et al. compared 155 normozoospermic men without varicocele and 363 men with varicocele, the latter in which both the site (left, right or bilateral) and grade (1, 2 or 3) were specified. Abnormal forms and sperm motility were worse in the higher grades, whereas no significant difference in concentration among the various groups were seen 14. Al-Ali et al. also found progressively worse semen parameters with higher grades of varicocele in 716 consecutive patients with varicocele 15. In our study, all semen variables including sperm concentration, motility, and strict normal morphology seemed to be lower with the increasing grade of varicocele in IUI cycles and were highest in the no varicocele group. Compared to the no varicocele group, grade 3 varicocele was negatively associated with sperm concentration and strict normal morphology. There was a higher prevalence of varicocele in men participating in ICSI than in IUI or IVF cycles, possibly due to more severe oligospermia or azoospermia. The same results seemed to be found in IVF, a decreasing trend was seen in all semen variables in men with grade 3 compared with the no varicocele group, but it did not reach statistical significance. Our results were in accordance with previous reports, implying that varicocele was one of the major causes of male infertility.

IUI is a fertility treatment that involves placing sperm inside a woman's uterus to facilitate fertilisation. The goal of IUI is to increase the number of sperms that reach the fallopian tubes and subsequently increase the chance of fertilisation. The amount of active, healthy sperm in a man's semen sample is often one of the biggest influences on IUI success 16. In general, IUI performed with a good-average to high sperm count carries a success rate between 15% and 20% per cycle, but there does not seem to be much difference in success rates between men with good-average sperm counts and men who have high sperm counts. It is not surprising that the higher the sperm count, the more likely it is that the procedure will be successful 17,18. In our study, men with grade 3 showed lower sperm parameters than those in the no varicocele group. Varicocele was negatively associated with all assessed semen variables except for sperm motility. Men with grade 3 varicocele had a lower sperm concentration and strict normal morphology than those in the no varicocele group, and a lower rate of pregnancy and live births was seen when male partners had a grade 3 varicocele versus those with no varicocele. Higher sperm motility was seen in men who originated pregnancies and live births (52.63±9.58%) than in those who did not (48.94±9.19%, *P*<0.005). Furthermore, this study revealed that the OR of pregnancy or live birth in men with grade 3 varicocele participating IUI decreased 31.9-45.2% or 12.5-47.3% compared with grades 1 and 2, respectively. Our observations also suggested that the increasing grade of varicocele might result in a lower semen quality and a lower IUI success.

Polackwich 19 evaluated the effects of the presence of varicocele on IVF outcomes. A total of 194 cycles were performed. Of those, 11, 54, 40, and 89 originated from men with grade 3, 2, 1, and no varicocele, respectively. There were no statistically significant differences in semen parameters. Comparing men with varicocele to those without, the former had a higher number of embryos transferred but less embryos frozen. This suggests varicocele might alter the outcomes, but significant differences were not seen. In our study, the chance of live birth when men had grade 3 varicocele was significantly lower and the miscarriage rate was higher than that of men without varicocele in IVF. Furthermore, the ORs for live birth in IVF were significantly lower when male partners had grade 3 varicocele as compared to those with a grade lower than 3. Although none of the classical semen parameters including sperm concentration, motility, and morphology were found to be associated with the outcomes of IVF treatment in this study, we could not exclude the fact that the increasing grade of varicocele and its association with the increased production of reactive oxygen species (ROS) reduced the total antioxidant capacity of semen or increased DNA damage in sperm, which has been recognized as one of the important determinants of normal fertilisation and embryo growth in natural and assisted conception 8,20. Saleh et al. 21 found that sperm DNA fragmentation was significantly increased in patients with infertility and varicocele in comparison to patients with normal results on genital examination. Elevated levels of sperm DNA fragmentation have been significantly associated with a negative pregnancy outcome including an increased time for conception, impaired embryo cleavage, higher miscarriage rates, and recurrent pregnancy loss after IVF 22, 23.

In this study, we found that ICSI had different results from IUI/IVF, with a comparable outcome on pregnancy or live birth rates when males had grades 1, 2, or 3 varicocele when compared to the no varicocele group. The explanation behind the superior results of ICSI might be due to different culture environments used for these techniques. While IVF oocytes were exposed to spermatozoa for 90 min, in ICSI, the spermatozoon were injected directly into the oocyte. The oocyte could, therefore, be less exposed to ROS in ICSI than in IVF. Secondly, women undergoing ICSI, on average, produce healthier oocytes with a better capacity for repair of the damaged sperm than women undergoing IVF, as in the ICSI group, infertility is mainly caused by males 22,23. In addition, we found a higher live birth in ICSI than IVF when male partners were diagnosed with grade 3 varicocele within the same group (50.8% vs 25.9%). Whether or not the superiority of ICSI oocytes should be recommended in the presence of a high grade 3 varicocele when natural conception is not possible despite excellent female fertility needs further research such as a randomised controlled trial between IVF and ICSI with men with grade 3 varicocele.

This study is the largest ever-reported study on the grades of varicocele in relation to the outcome of ART, demonstrating that the increasing varicocele grade of is negatively associated with sperm parameters and can alter the outcome of further IUI/IVF. However, our study had a few limitations. The population size of the patients with high grade 3 varicocele was relatively small, especially in IUI treatment, where no cases of miscarriage and ectopic pregnancy were found when the male partner had grade 3 varicocele. Future studies should involve a larger cohort required to detect a statistically significant difference in miscarriage and ectopic outcomes.

## Figures and Tables

**Table 1:** Varicocele data

	IUI (n=1501)	IVF (n=1623)	ICSI (n=1079)
Number (%)	311	294	286
**Varicocele, side, no. (%)**	20.7%	18.1%	26.5%
Unilateral, left	294	240	101
Unilateral, right	-	-	-
Bilateral	17	6	14
Total	311	246	115
**Varicoceles, grade, no. (%)**			
Grade 1	166	157	155
Grade 2	80	79	72
Grade 3	65	58	59
Total	56	255	126

IUI: intrauterine insemination; IVF: *in vitro* fertilization; ICSI: intracytoplasmic sperm injection.

**Table 2:** Demographic data of IUI cycles

	All	Grade 1	Grade 2	Grade 3	No varicocele	*p*
Number (%)	1501	166 (11.06%)	80 (5.33%)	65 (4.33%)	1190 (79.28%)	
Primary/secondary	1092/409 (72.8%)	125/41 (75.3%)	58/22 (72.5%)	49/16 (75.4%)	860/33 0 (72.3%)	0.838
Male age (years)	30.93±4.84	31.00±4.15	30.92±3.49	31.66±3.79	30.38±4.76	0.141
Male BMI (kg/m^2^)	23.28±4.40	23.61±5.16	22.35±3.47	25.13±3.82	22.83±3.72	0.063
Female age (years)	29.87±3.92	29.78±3.28	29.79±3.42	31.83±3.71	29.55±4.31	0.326
Female b-FSH (IU/l)	6.88±2.05	6.85±1.24	6.30±2.71	6.89±1.18	6.81±1.97	0.594
**Sperm**						
Concentration (M/mL)	70.95±36.58	70.12±34.91*	67.10±41.15	49.39±15.83*	74.94±38.49	0.071
Motility (%)	58.00±16.27	58.67±15.65	55.16±15.14	53.21±19.88	58.61±16.44	0.522
Normal morphology (%)	3.22±1.51	3.51±1.41*	3.01±1.62	2.31±0.78*	3.11±1.61	0.262
**Outcome**						
Rate of pregnancy (%)	211	23 (13.8%)	13 (16.2%)	3 (4.6%)*	172 (14.4%)	0.440
Rate of miscarriage (%)	27	5 (3.0%)	2 (2.5%)	0	20 (1.7%)	0.424
Rate of ectopic (%)	0	0	0	0	4 (0.3%)	NA
Rate of live birth (%)	180	18 (10.8%)	11 (13.8%)	3 (4.6%)	148 (12.4)	0.26

BMI: body mass index; FSH: follicle-stimulating hormone; *: *p*<0.05, compared with the no varicocele group.

**Table 3:** Data of pregnancy, miscarriage, ectopic, and live births in IUI, IVF and ICSI cycles in male with grade 3 varicocele versus the no varicocele group

	IUI Grade 3	No varicocele	OR (95% CI)	*p*	IVF Grade 3	No varicocele	OR (95% CI)	*p*	ICSI Grade 3	No varicocele	OR (95% CI)	*p*
Cycles (n)	65	1190	—	—	58	1329	—	—	59	793	—	—
Pregnancy % (n)	4.6% (3)	14.4% (172)	0.600 (0.400-0.900)	0.013	41.4 (24)	52.4 (697)	0.883 (0.707-1.073)	0.212	59.3 (35)	56.0 (444)	1.115 (0.897-1.385)	0.327
Miscarriage % (n)	0	1.7% (27)	0.004 (0.00-NO)	0.997	13.8 (8)	7.5 (100)	1.469 (1.152-1.874)	0.002	6.8 (4)	4.9 (39)	1.262 (0.880-1.811)	0.206
Ectopic rate % (n)	0	0.17% (2)	0.003 (0.00-NO)	0.997	1.7 (1)	2.7 (36)	1.210 (0.730-2.003)	0.460	1.7 (1)	1.3 (10)	1.372 (0.678-2.775)	0.379
Live birth % (n)	4.6% (3)	12.4% (148)	0.649 (0.433-0.975)	0.037	25.9 (15)	42.2 (561)	0.690 (0.544-0.874)	0.002	50.8 (30)	49.8 (395)	1.025 (0.831-1.265)	0.815

IUI: intrauterine insemination; IVF: *in vitro* fertilisation; ICSI: intracytoplasmic sperm injection.

**Table 4:** Demographic data of IVF cycles

	All	Grade 1	Grade 2	Grade 3	No-Varicocele	*p*
Number (%)	1623	157 (9.67%)	79 (4.87%)	58 (3.57%)	1329 (81.9%)	
Primary/secondary	692/931	62/95 (39.5%)	36/43 (45.6%)	25/33 (43.1%)	569/760 (42.8%)	0.817
Male age (years)	32.91±4.52	32.84±4.25	32.64±4.66	32.03±4.24	33.12±4.72	0.218
Male BMI (kg/m^2^)	23.21±2.81	23.23±2.73	24.16±2.82	23.18±2.34	23.03±2.91	0.092
Female age (years)	31.24±4.04	31.52±3.97	30.64±3.76	30.27±4.24	31.28±4.09	0.803
Female b-FSH (IU/l)	6.73±1.67	6.77±1.65	6.43±1.74	6.81±1.47	6.74±1.69	0.855
**Sperm**						
Concentration (M/mL)	77.92±58.08	81.87±48.15	68.34±42.81	65.34±36.99	78.66±68.33	0.640
Motility (%)	56.90±16.94	57.18±16.95	53.40±16.27	55.13±19.03	57.58±16.70	0.303
Normal morphology (%)	2.16±1.39	2.18±1.39	1.88±1.33	1.85±1.02	2.22±1.44	0.21
**Outcome**						
Oocytes retrieved (n)	11.29±6.76	11.53±7.06	12.46±7.12	11.51±5.83	10.82±6.49	0.037
2 pronuclei (n)	7.37±4.32	7.67±4.38	7.42±4.30	7.27±4.33	7.46±4.28	0.525
No. of good embryos	3.70±3.01	3.90±3.00	3.66±3.29	3.42±3.36	3.54±2.92	0.125
Embryos transferred (n)	2.19±0.55	2.20±0.53	2.15±0.56	2.00±0.45	2.19±0.56	0.793
Rate of fertilisation (%)	51.44±33.51	56.30±34.04	58.68±34.94	46.36±32.54	58.65±34.84	0.422
Rate of good embryos (%)	34.44±22.77	36.27±22.91	39.73±25.92	33.56±24.42	34.26±22.75	0.610
Rate of pregnancy (%)	832	75 (47.8%)	36 (45.6%)	24 (41.3%)	697 (52.4%)	0.980
Rate of miscarriage (%)	121	9 (5.7%)	4 (5.1%)	8 (13.7%)	100 (7.5%)	0.126
Rate of ectopic (%)	39	1 (0.6%)	1 (1.3%)	1 (1.7%)	36 (2.7%)	0.935
Rate of Live birth (%)	672	65 (41.4%)	31 (39.2%)	15 (25.9%)*	561 (42.2%)	0.322

BMI: body mass index; FSH: follicle-stimulating hormone; *: *p*<0.05, compared with the no varicocele group.

**Table 5:** Demographic data of ICSI cycles

	All	Grade 1	Grade 2	Grade 3	No-Varicocele	*p*
Number (%)	1079	155 (14.4%)	72 (6.67%)	59 (5.47%)	793 (73.4%)	
Primary/secondary	881/198	125/30 (80.6%)	63/9 (87.5%)	50/9 (84.7%)	643/150 (81.1%)	0.513
Male age (years)	31.18±4.50	31.14±4.47	30.47±3.76	32.27±4.62	31.17±4.60	0.984
Male BMI (kg/m^2^)	23.12±2.72	22.68±2.66	22.75±2.81	21.81±2.33	22.62±2.78	0.948
Female age (years)	29.77±4.06	29.72±4.09	29.91±3.67	30.86±3.41	29.64±4.16	0.422
Female b-FSH (IU/l)	6.58±1.60	6.55±1.66	6.30±1.02	6.61±1.77	6.64±1.60	0.324
**Sperm**						
Concentration (M/mL)	16.61±28.17	15.75±26.30	25.68±36.22	15.49±24.92	16.02±28.31	0.585
Motility (%)	19.15±22.42	19.90±22.69	23.12±21.57	22.95±24.97	17.29±22.07	0.029
Normal morphology (%)	0.39±0.71	0.44±0.72	0.45±0.65	0.32±0.64	0.35±0.71	0.157
**Outcome**						
Oocytes retrieved (n)	12.25±5.31	12.06±5.32	12.69±5.51	11.45±5.03	12.52±5.48	0.506
2 pronuclei (n)	8.90±4.33	8.79±4.34	8.12±3.97	9.53±4.59	9.00±4.63	0.780
No. of good embryos	3.78±3.29	3.92±3.35	4.25±3.45	3.61±3.23	3.72±3.51	0.844
Embryos transferred (n)	1.99±0.36	1.99±3.51	2.00±0.41	2.02±0.35	1.99±0.40	0.887
Rate of fertilisation (%)	72.81±19.54	73.57±19.29	71.89±21.22	75.48±16.92	72.07±19.82	0.344
Rate of good embryos (%)	41.07±63.89	44.77±76.71	26.77±26.58	46.67±49.88	40.06±59.84	0.669
Rate of pregnancy (%)	621	94 (60.6%)	48 (66.7%)	35 (59.3%)	444 (56.0%)	0.178
Rate of miscarriage (%)	62	12 (7.7%)	7 (9.7%)	4 (6.8%)	39 (4.9%)	0.090
Rate of ectopic (%)	16	4 (2.6%)	1 (1.4%)	1 (1.7%)	10 (1.3%)	0.433
Rate of Live birth (%)	543	78 (50.3%)	40 (55.6%)	30 (50.8%)	395 (49.8%)	0.832

BMI: body mass index; FSH: follicle-stimulating hormone.

## Abbreviations

AST: assisted reproductive technology
IUI: intrauterine semination
IVF: invitro fertilisation
ICSI: intracytoplasmic sperm injection
OR: odds ratio
BMI: body mass index
FSH: follicle-stimulating hormone
CI: confidence interval
ROS: reactive oxygen species
